# Extent and Progression of Cardiac Damage in Patients With Primary Mitral Regurgitation Undergoing Surgical Repair

**DOI:** 10.1093/ejcts/ezag149

**Published:** 2026-04-10

**Authors:** Rinchyenkhand Myagmardorj, Federico Fortuni, Valentina Mantegazza, Aniek L van Wijngaarden, Hoi Wai Wu, Meindert Palmen, Mauro Pepi, Laura Fusini, Manuela Muratori, Benjamin Essayagh, Christophe Tribouilloy, Thierry Le Tourneau, Yohann Bohbot, Hector I Michelena, Maurice Enriquez-Sarano, Jeroen J Bax, Nina Ajmone Marsan

**Affiliations:** Department of Cardiology, Heart Lung Center, Leiden University Medical Center, Leiden 2333 ZA, The Netherlands; Department of Cardiology, Heart Lung Center, Leiden University Medical Center, Leiden 2333 ZA, The Netherlands; Cardiology and Cardiovascular Pathophysiology, S. Maria Della Misericordia Hospital, University of Perugia, Perugia 06129, Italy; Department of Peri-Operative Cardiology and Cardiovascular Imaging, Centro Cardiologico Monzino IRCCS, Milan 20138, Italy; Department of Cardiology, Heart Lung Center, Leiden University Medical Center, Leiden 2333 ZA, The Netherlands; Department of Cardiology, Heart Lung Center, Leiden University Medical Center, Leiden 2333 ZA, The Netherlands; Department of Cardiothoracic Surgery, Leiden University Medical Center, Leiden 2333 ZA, The Netherlands; Department of Peri-Operative Cardiology and Cardiovascular Imaging, Centro Cardiologico Monzino IRCCS, Milan 20138, Italy; Department of Peri-Operative Cardiology and Cardiovascular Imaging, Centro Cardiologico Monzino IRCCS, Milan 20138, Italy; Department of Peri-Operative Cardiology and Cardiovascular Imaging, Centro Cardiologico Monzino IRCCS, Milan 20138, Italy; Department of Cardiovascular Medicine, Mayo Clinic, Rochester, MN 55905, United States; Department of Echocardiography, Cardio X Clinic, Cannes 06400, France; Department of Cardiology, Amiens University Hospital, Amiens 80054, France; l’Institut du Thorax, Institut national de la santé et de la recherche médicale, Centre national de la recherche scientifique, Université de Nantes, Nantes 44000, France; Department of Cardiology, Amiens University Hospital, Amiens 80054, France; Department of Cardiovascular Medicine, Mayo Clinic, Rochester, MN 55905, United States; Department of Cardiovascular Medicine, Allina Health Minneapolis Heart Institute—Abbott Northwestern Hospital, Minneapolis, MN 55407, United States; Department of Cardiology, Heart Lung Center, Leiden University Medical Center, Leiden 2333 ZA, The Netherlands; Department of Cardiology, Heart Lung Center, Leiden University Medical Center, Leiden 2333 ZA, The Netherlands

**Keywords:** primary mitral regurgitation, mitral valve annuloplasty, mitral valve prolapse

## Abstract

**Objectives:**

The progression of extra-mitral valve (MV) cardiac damage in patients with primary mitral regurgitation (MR) following surgical repair has not been described. We aimed to investigate the evolution of extra-MV cardiac damage after MV repair and to assess its prognostic significance.

**Methods:**

Patients with severe primary MR undergoing surgical repair at 5 referral centres were included when echocardiographic follow-up assessment was available. Based on echocardiographic parameters at baseline and at a median follow-up of 7 (interquartile range [IQR] 4-11) months after MV repair, patients were hierarchically classified as follows: stage 0: no cardiac damage; stage 1: left ventricular dilatation and/or dysfunction; stage 2: left atrial dilatation and/or atrial fibrillation; stage 3: pulmonary hypertension and/or significant tricuspid regurgitation. The primary outcome was all-cause mortality.

**Results:**

A total of 764 patients (mean age 62 ± 13 years, 70% men) were included. Compared to baseline, 43% improved at least 1 stage, 49% remained unchanged, and 8% worsened at least 1 stage during follow-up after MV repair. At a median follow-up of 91 months after MV repair, 94 patients (12%) died. By multivariate Cox regression analysis, after adjusting for potential confounders, extra-MV cardiac damage staging at follow-up (HR per-1-stage-increase = 1.571; *P* = .009) and cardiac damage evolution (improved group, HR = 0.467; *P* = .034; worsened group, HR = 2.481, *P* = .037) were independently associated with all-cause mortality and had incremental prognostic value over preprocedural assessment.

**Conclusions:**

Extra-MV cardiac damage improves significantly after MV repair, and its evolution is independently associated with all-cause mortality, suggesting the importance of comparative echocardiographic assessment following MV repair to improve risk stratification.

## Graphical abstract

**Figure:**
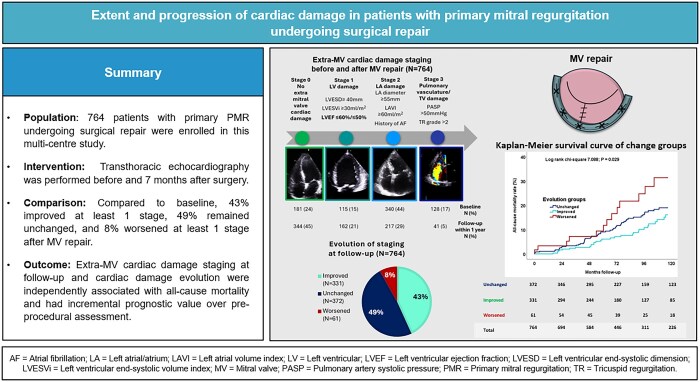

## Introduction

Primary mitral regurgitation (PMR) is the second most prevalent valvular heart disease worldwide after degenerative aortic stenosis.^1^ When significant (ie, ≥ moderate), PMR is associated with high morbidity and mortality, and treatment typically involves either surgical mitral valve (MV) repair or transcatheter interventions.^2^^,^^3^

For the management of patients with severe PMR, current guidelines recommend evaluating both clinical and key imaging parameters to guide treatment decisions, their timing, and risk stratification.^2^^,^^3^ These parameters include symptoms, left ventricular (LV) size and function, pulmonary artery systolic pressure (PASP), and the presence of atrial fibrillation (AF). More recently, additional prognostic markers such as concomitant tricuspid regurgitation (TR), left atrial volume index (LAVI), and right ventricular (RV) function have been recognized as important contributors to patient outcomes.^4–6^ Successful interventions for PMR have been shown to result in significant improvements in LV size, LAVI, TR severity, pulmonary pressures, and RV function.^7^^,^^8^ Therefore, after surgery, these patients present an overall different cardiac profile, whose potential impact on long-term prognosis has never been tested. In this multicentre observational study, we applied a previously proposed^9^ staging approach to assess extra-MV cardiac damage both before and after surgical MV repair. Our aim was to characterize the overall structural and functional cardiac changes following surgical MV repair and to evaluate the prognostic significance of cardiac damage evolution and post-repair staging in comparison with preprocedural assessment.

## Methods

### Patient population

Patients with at least moderate-to-severe primary MR undergoing surgical MV repair at 5 referral centres, including Leiden University Medical Center (Leiden, NL), Centro Cardiologico Monzino (Milan, IT), Mayo Clinic (Rochester, Minnesota, USA), Nantes University Hospital (Nantes, FR), and Amiens University Hospital (Amiens, FR), between 2000 and 2021 were retrospectively included based on the availability of transthoracic echocardiography (TTE) at baseline and at follow-up after surgery (between 3 and 12 months).

The indication for MV surgery was based on local heart team assessment. The following exclusion criteria were applied: MV replacement, infective endocarditis, rheumatic heart disease, significant aortic valve disease, and previous valvular interventions.

Baseline demographic and clinical data of all study participants were collected from hospital records prior to surgery. This included patients’ history of concomitant comorbidities, cardiovascular risk factors, New York Heart Association (NYHA) functional class, medications, and the latest serum creatinine level. Clinical follow-up data were also obtained from local hospital records. Mitral valve surgical repair techniques were thoroughly explained previously^10^^,^^11^ and included chordal replacement for anterior MV leaflet prolapse, papillary muscle head repositioning for commissural prolapse, and a combination of resection and neochord techniques was performed for the posterior MV leaflet, separately. In all cases, for stabilizing MV annulus and the suture line, a ring annuloplasty without downsizing was performed. The current study was approved by the local ethics committee of referral centres (approval number: CME 10/024/SH; CME 10/025/SH) and conducted in accordance with the Declaration of Helsinki. Due to the retrospective design of this study, the Medical Ethical Committee waived the need of written informed consent.

### TTE assessment

A comprehensive TTE assessment was performed before surgery (median 38 days [interquartile range (IQR) 7-119 days] before the MV repair) and repeated at a median follow-up of 7 (IQR 4-11) months using commercially available machines (GE Healthcare: Vivid 5, 7, E9, and E95; Philips Medical Systems: IE33 and Epiq). Digitally stored offline echocardiographic images were then retrospectively analysed by experienced cardiologists. Left ventricular linear dimensions were measured from the parasternal long-axis view, and Devereux’s formula was used to derive LV mass.^12^ The apical 4- and 2-chamber views were used to derive LV volumes and left ventricular ejection fraction (LVEF) with the biplane Simpson volumetric method. Maximal LA diameter was assessed from parasternal long-axis view at end-systole.^12^ The apical 4- and 2-chamber views were used to derive the biplane LA maximum volume, which was indexed to body surface area.^12^ The severities of MR and TR were assessed and graded with a multi-parametric approach according to current guidelines.^13^ PASP was derived by adding right atrial pressure (estimated based on inferior vena cava diameter and collapsibility) to TR peak pressure gradient.^14^ Right ventricular systolic function was assessed based on tricuspid annular plane systolic excursion (TAPSE).^12^

### Extra-MV cardiac damage staging

**Figure 1.**
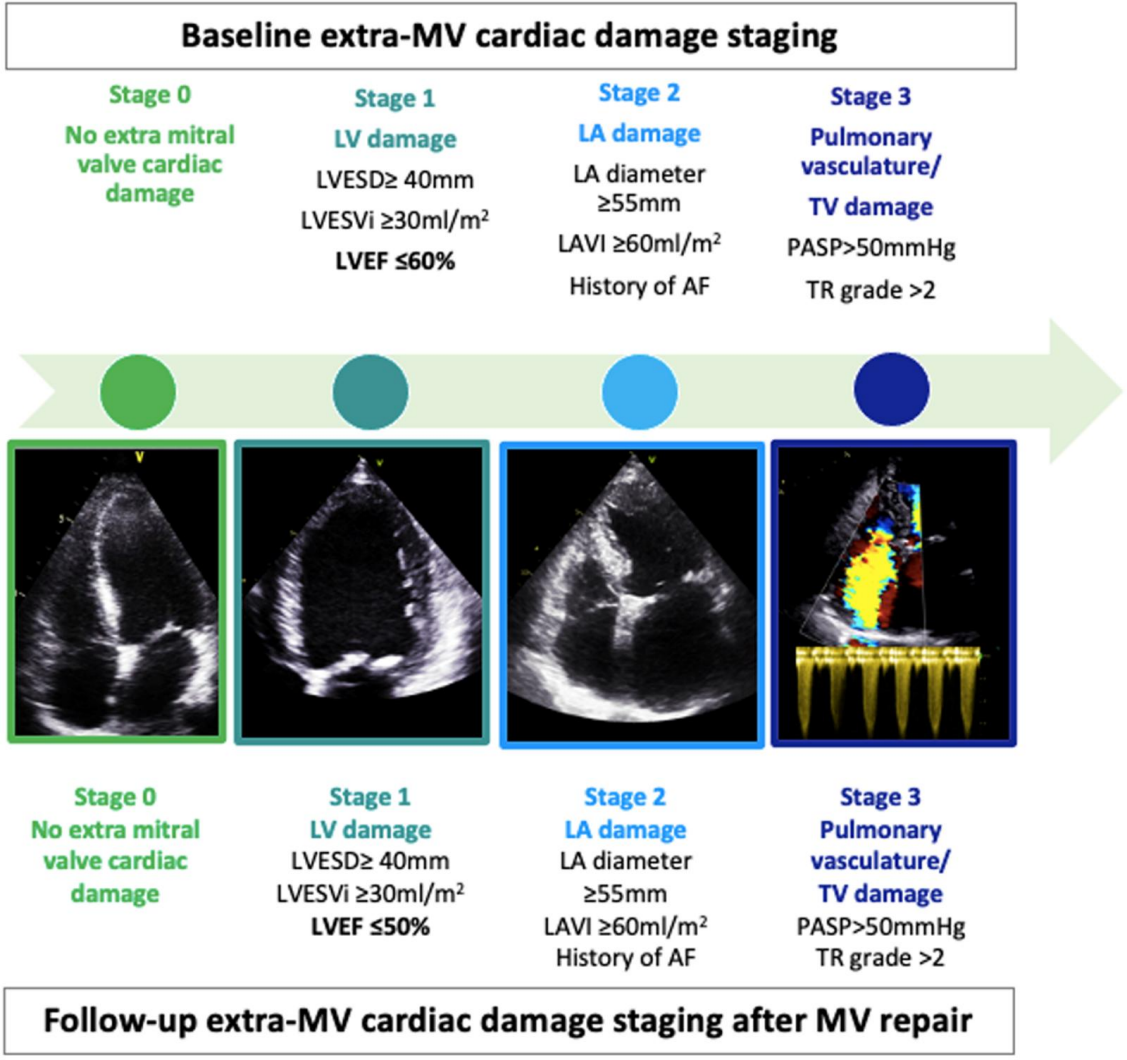
The Severity of Extra-MV Cardiac Damage Stages. Abbreviations: AF, atrial fibrillation; LA, left atrial/atrium; LAVI, left atrial volume index; LV, left ventricular; LVEF, left ventricular ejection fraction; LVESD, left ventricular end-systolic dimension; LVESVi, left ventricular end-systolic volume index; MV, mitral valve; PASP, pulmonary artery systolic pressure; TR, tricuspid regurgitation.**Alt Text**: The graph showed echocardiography based criteria included in 4 different stages used both at baseline and follow-up.

Patients were hierarchically classified into the following stages of extra-MV cardiac damage based on baseline echocardiographic assessment as previously proposed^9^^,^^15^ (**Figure 1**): stage 0: no signs of extra-MV cardiac damage; stage 1: LV damage which was defined based on LV dimensions (LV end-systolic diameter [LVESD] ≥ 40 mm and/or LV end-systolic volume index [LVESVi] ≥ 30 mL/m^2^) and/or systolic dysfunction (LVEF ≤ 60%); stage 2: LA damage identified based on LA dimensions (LA diameter ≥ 55 mm and/or LAVI ≥ 60 mL/m^2^) and/or history of AF; stage 3: pulmonary vasculature or tricuspid valve (TV) damage which was identified based on PASP >50 mmHg and/or TR grade >2. At follow-up, patients were then reclassified in the same stages based on the new echocardiographic assessment. However, the cut-off value for LVEF to define LV systolic dysfunction (stage 1) after MV repair was set at 50%, as this threshold more accurately reflects impaired systolic function in the context of increased LV afterload following successful MV repair.^15–17^ Importantly, unlike previously proposed cardiac damage staging systems, RV dysfunction assessed with TAPSE was not included in our assessment of cardiac damage at either baseline or follow-up. This decision was based on the well-documented postoperative decline in TAPSE, particularly when TV repair is also performed, which limits its reliability as a marker of true RV dysfunction and therefore for risk stratification.^4^^,^^7^^,^^8^^,^^18^

### Study outcome

The study end-point was all-cause mortality, which was assessed based on local hospital systems and governmental death registries. Follow-up data were complete for all patients.

### Statistical analysis

Continuous variables were expressed as mean (standard deviations) (if normally distributed) or median with [IQR]. Categorical variables were presented as absolute numbers and percentages (%) and compared using the Chi-square test for between groups comparison and the McNemar test for paired comparison. When comparing continuous variables, 1-way analysis of variance (ANOVA) or Kruskal-Wallis test (with Bonferroni correction) were used for between groups comparisons, and paired t-test or Wilcoxon paired rank test were used for paired analysis.

Survival analysis was performed using Kaplan-Meier curves with log-rank test which was used for comparing the cumulative event rates between stage groups. A landmark analysis was performed to evaluate the association between extra-MV cardiac damage assessed at follow-up after surgery and the 10-year outcomes.^19^ Univariable Cox regression analyses were used to evaluate the association of clinical variables, echocardiographic parameters, and extra-MV cardiac damage with the primary end-point. Statistically significant covariates at the univariable analysis or clinically relevant variables were chosen to be included into different multivariable Cox regression models. Hazard ratios (HRs) and 95% confidence intervals (CIs) were calculated and presented.

Two-sided *P*-values less than .05 were considered as statistically significant. Statistical analysis was performed using SPSS version 29.0 (IBM Corporation).

## Results

### Baseline and follow-up extra-MV myocardial damage

A total of 1832 patients underwent MV repair between 2000 and 2021 at our centres. Of those, 764 patients were included in this analysis after applying the exclusion criteria (**Figure S1**). As shown in the **Table S1**, the included and excluded cohorts were similar limiting the risk of selection bias. Before MV repair, a total of 181 patients (24%) were classified in stage 0, while 115 patients (15%) in stage 1, 340 (44%) in stage 2, and 128 (17%) in stage 3. **Table 1** shows the baseline clinical characteristics according to the extra-MV cardiac damage before MV repair. In the per-group analysis, patients with more advanced extra-MV cardiac damage before surgery were older, presented with more advanced heart failure (HF) symptoms, had a higher comorbidity burden, including diabetes mellitus, AF, and underwent more frequently concomitant TV repair and MAZE procedure.

**Table 1.** Baseline Clinical Characteristics of the Study Population Divided According to the Extra-MV Cardiac Damage Assessed Before MV Surgery (*n* = 764)

	Baseline staging				*P*-value
	Stage 0 (*n* = 181, 24%)	Stage 1 (*n* = 115, 15%)	Stage 2 (*n* = 340, 44%)	Stage 3 (*n* = 128, 17%)	
Age, years	60.1 (11.7)	58.1 (14.6)	61.1 (12.4)	67.8 (10.3)	**<.001**
Male gender, *n* (%)	127 (70)	87 (76)	243 (72)	79 (62)	.098
Body mass index (kg/m^2^)	24.9 (3.6)	25.4 (3.6)	24.9 (3.8)	25.2 (4.0)	.515
Body surface area (m^2^)	1.9 (0.2)	2.0 (0.2)	1.9 (0.2)	1.9 (0.2)	**.010**
Hypertension, *n* (%)	64 (37)	37 (34)	107 (37)	54 (47)	.159
Diabetes mellitus, *n* (%)	6 (3)	3 (3)	5 (2)	9 (7)	**.019**
Coronary artery disease, *n* (%)	33 (19)	23 (22)	52 (18)	34 (31)	**.047**
Atrial fibrillation, *n* (%)	0 (0)	0 (0)	148 (44)	45 (35)	**<.001**
History of smoking, *n* (%)	53 (34)	29 (30)	90 (34)	34 (35)	.884
Chronic obstructive pulmonary disease, *n* (%)	7 (5)	4 (5)	10 (4)	6 (6)	.646
NYHA class II or more	112 (66)	76 (69)	234 (72)	102 (90)	**.001**
Creatinine	1.0 (0.2)	1.0 (0.3)	1.0 (0.4)	1.1 (0.4)	.253
eGFR, mL/min//1.73 m	77.9 (19.5)	78.6 (24.7)	79.1 (24.2)	66.3 (18.9)	**<.001**
Concomitant surgery					
CABG	29 (17)	14 (13)	32 (11)	22 (19)	.125
Tricuspid valve surgery	34 (19)	29 (25)	120 (35)	47 (37)	**<.001**
MAZE procedures	7 (4)	6 (6)	91 (34)	32 (32)	**<.001**

Values are mean (SD), or *n* (%). Statistical significance at the .05 level is shown in bold type.

Abbreviations: CABG, coronary artery bypass graft; eGFR, estimated glomerular filtration rate; NYHA, New York Heart Association.

**Table 2** shows the baseline echocardiographic characteristics according to the extra-MV cardiac damage stages before MV repair. Overall, there were progressive worsening in echocardiographic parameters as the stage of extra-MV cardiac damage increased. In the per-group comparison, patients with more advanced extra-MV cardiac damage presented with increased LV mass, enlarged LV and LA volumes, increased PASP, and with a higher prevalence of significant TR as well as RV systolic dysfunction.

**Table 2.** Baseline Echocardiographic Characteristics (*n* = 764) of the Study Divided According to the Extra-MV Cardiac Damage Assessed Before MV Surgery

	Baseline staging				*P*-value
	Stage 0 (*n* = 181, 24%)	Stage 1 (*n* = 115, 15%)	Stage 2 (*n* = 340, 44%)	Stage 3 (*n* = 128, 17%)	
LVEDD, mm	53.5 (5.6)	57.8 (6.6)	57.6 (7.2)	56.5 (7.0)	**<.001**
LVESD, mm	31.0 (4.8)	38.1 (7.0)	35.9 (6.9)	34.9 (6.0)	**<.001**
LVESD ≥ 40, *n* (%)	0 (0)	55 (49)	97 (29)	29 (23)	**<.001**
LV mass index, g/m^2^	114.0 (25.8)	121.7 (24.1)	130.6 (29.1)	131.4 (28.5)	**<.001**
LVEDV index, mL/m^2^	69.0 (15.5)	76.7 (22.6)	85.0 (25.8)	79.9 (24.3)	**<.001**
LVESV index ≥ 30 mL/m^2^, *n* (%)	0 (0)	41 (51)	119 (44)	25 (31)	**<.001**
LVEF, %	68.9 (4.6	59.8 (6.8	64.3 (8.6	66.0 (7.3	**<.001**
LVEF < 60%, *n* (%)	0 (0)	66 (58)	91 (27)	21 (17)	**<.001**
LA diameter, mm	41.3 (5.5)	42.3 (5.0)	47.2 (7.4)	49.5 (7.9)	**<.001**
LA diameter ≥ 55 mm, *n* (%)	0 (0)	0 (0)	44 (16)	16 (19)	**<.001**
LA volume index, mL/m^2^	44.4 (9.4)	44.6 (9.4)	73.6 (26.4)	70.1 (23.7)	**<.001**
LA volume index ≥ 60 mL/m^2^, *n* (%)	0 (0)	0 (0)	263 (77)	77 (62)	**<.001**
PASP, mmHg	30.2 (6.9)	30.0 (7.3)	34.5 (7.9)	58.1 (13.0)	**<.001**
PASP > 50 mmHg, *n* (%)	0 (0)	0 (0)	0 (0)	102 (78)	**<.001**
TR grade > grade 2, *n* (%)	0 (0)	0 (0)	0 (0)	38 (30)	**<.001**
TAPSE, mm	24.6 (4.0)	23.5 (4.0)	24.0 (5.1)	22.3 (4.9)	**.004**
TAPSE ≤17 mm, *n* (%)	1 (1)	5 (6)	24 (9)	18 (20)	**<.001**

Values are mean (SD) or *n* (%). Statistical significance at the .05 level is shown in bold type.

Abbreviations: LA, left atrial/atrium; LV, left ventricular; LVEDD, left ventricular end-diastolic dimension; LVEDV, left ventricular end-diastolic volume; LVEF, left ventricular ejection fraction; LVESD, left ventricular end-systolic dimension; LVESV, left ventricular end-systolic volume; PASP, pulmonary artery systolic pressure; TAPSE, tricuspid annular plane systolic excursion; TR, tricuspid regurgitation.

Echocardiographic follow-up was conducted at a median of 7 months [IQR: 4-11 months] after the procedure. In comparison to the baseline assessment, after MV repair, most echocardiographic parameters improved; LV and LA dimensions significantly decreased, as well as mean PASP (37 vs 26 mmHg) and the prevalence of severe TR (5% vs 3%). However, LV systolic function assessed with LVEF decreased (65% vs 56%) as well as RV systolic function assessed with TAPSE (24 mm vs 18 mm) (**Table 3**).

**Table 3.** Paired Comparison of Baseline and Follow-Up Echocardiographic Parameters and Extra-MV Cardiac Damage Staging Criteria (*n* = 764)

Variables		Baseline	Follow-up	*P*-value
LVEDD, mm		56.5 (7.0)	49.2 (6.6)	**<.001**
LVESD, mm		34.7 (6.7)	33.7 (6.8)	**<.001**
LV mass index, g/m^2^		125.1 (28.4)	104.7 (30.9)	**<.001**
LVEDV index, mL/m^2^		77.6 (19.2)	56.4 (15.1)	**<.001**
LVEF, %		65.1 (7.8)	55.8 (8.3)	**<.001**
LVEF ≤ 60%, *n* (%)		172 (23)	539 (73)	**<.001**
LA diameter, mm		45.5 (7.5)	39.9 (7.4)	**<.001**
LA volume index, mL/m^2^		62.0 (25.6)	40.6 (16.8)	**<.001**
PASP, mmHg		37.1 (13.3)	25.6 (11.3)	**<.001**
TAPSE, mm		23.6 (4.7)	17.7 (3.0)	**.020**
TAPSE ≤17 mm, *n* (%)		44 (9)	203 (40)	**<.001**
MR grade >2, *n* (%)		764 (100)	24 (3)	**<.001**
Follow-up staging criteria				
LVESD ≥ 40, *n* (%)	Stage 1	161 (27)	122 (18)	**.002**
LVESV index ≥ 30 mL/m^2^, *n* (%)		159 (31)	116 (23)	**<.001**
LVEF ≤ 50%, *n* (%)		26 (4)	167 (23)	**<.001**
LA diameter ≥ 55 mm, *n* (%)	Stage 2	56 (11)	25 (5)	**<.001**
LA volume index ≥ 60 mL/m^2^, *n* (%)		294 (46)	74 (12)	**<.001**
AF, *n* (%)		197 (25)	199 (25)	1.000
PASP > 50 mmHg, *n* (%)	Stage 3	96 (15)	19 (3)	**<.001**
TR grade > grade 2, *n* (%)		37 (5)	26 (3)	.161

Values are mean (SD) or *n* (%). Statistical significance at the .05 level is shown in bold type.

Abbreviations: AF, atrial fibrillation; LA, left atrial/atrium; LV, left ventricular; LVEDD, left ventricular end-diastolic dimension; LVEDV, left ventricular end-diastolic volume; LVEF, left ventricular ejection fraction; LVESD, left ventricular end-systolic dimension; LVESV, left ventricular end-systolic volume; MR, mitral regurgitation; PASP, pulmonary artery systolic pressure; TAPSE, tricuspid annular plane systolic excursion; TR, tricuspid regurgitation.

Based on the extra-MV cardiac damage definition, at follow-up after MV repair, 344 patients (45%) were classified in stage 0, 162 patients (21%) in stage 1, 217 patients (28%) in stage 2, and 41 patients (5%) in stage 3 (Central illustration, **Figure S2**). The comparison of baseline clinical and echocardiographic data according to the follow-up extra-MV cardiac damage staging is presented in **Tables S2** and **S3**. Based on these changes, the overall extent of extra-MV cardiac damage improved at follow-up after surgery (**Table 3**), and this was consistent also after excluding patients who did not receive concomitant myocardial revascularization (**Table S4**). In particular, 43% of the patients improved (improved group) by at least 1 cardiac damage stage (Central illustration). However, in 49% of the patients, extra-MV cardiac damage remained stable (unchanged group), and in 8% deteriorated (worsened group) after MV repair.

### Associates of the study end-point

**Figure 2.**
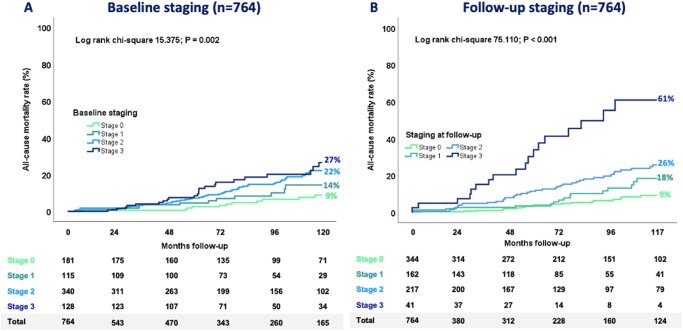
Kaplan-Meier Survival Curves for All-Cause Mortality According to the Baseline and Follow-Up Extra-MV Cardiac Damage Stages. Kaplan-Meier survival curves for all-cause mortality stratified according to the severity of staging before and after MV repair. Baseline staging curves start from the time of MV repair, while follow-up staging curve starts from the date of follow-up echo after MV repair (median 91 months; IQR 56-120 months).**Alt text: **Graphical representation of survival curves for mortality considering patients at stages 0 to 3 at baseline and follow-up.

During a median follow-up of 91 months after MV repair (IQR 56-120 months), 94 (12%) patients died. The survival rates were significantly different across the 4 stages of extra-MV cardiac damage, both when considering the baseline (log rank *P* = .002; Chi-square 15.375; **Figure 2A**) and the follow-up classification (log rank *P* = .001; Chi-square 75.110; **Figure 2B**). In particular, the 10-year all-cause mortality rates according to baseline cardiac damage were 9% for stage 0, 14% for stage 1, 22% for stage 2, and 27% for stage 3 (**Figure 2A**), whereas they were 9% for stage 0, 18% for stage 1, 26% for stage 2, and 61% for stage 3 (**Figure 2B**) according to the follow-up assessment. Regarding the evolution of cardiac damage following MV repair, patients with improved extra-MV cardiac damage had the highest survival rates, whereas those in the worsened group exhibited the poorest outcomes (log-rank *P* = .029; Chi-square = 7.088; see Central illustration).

**Figure 3.**
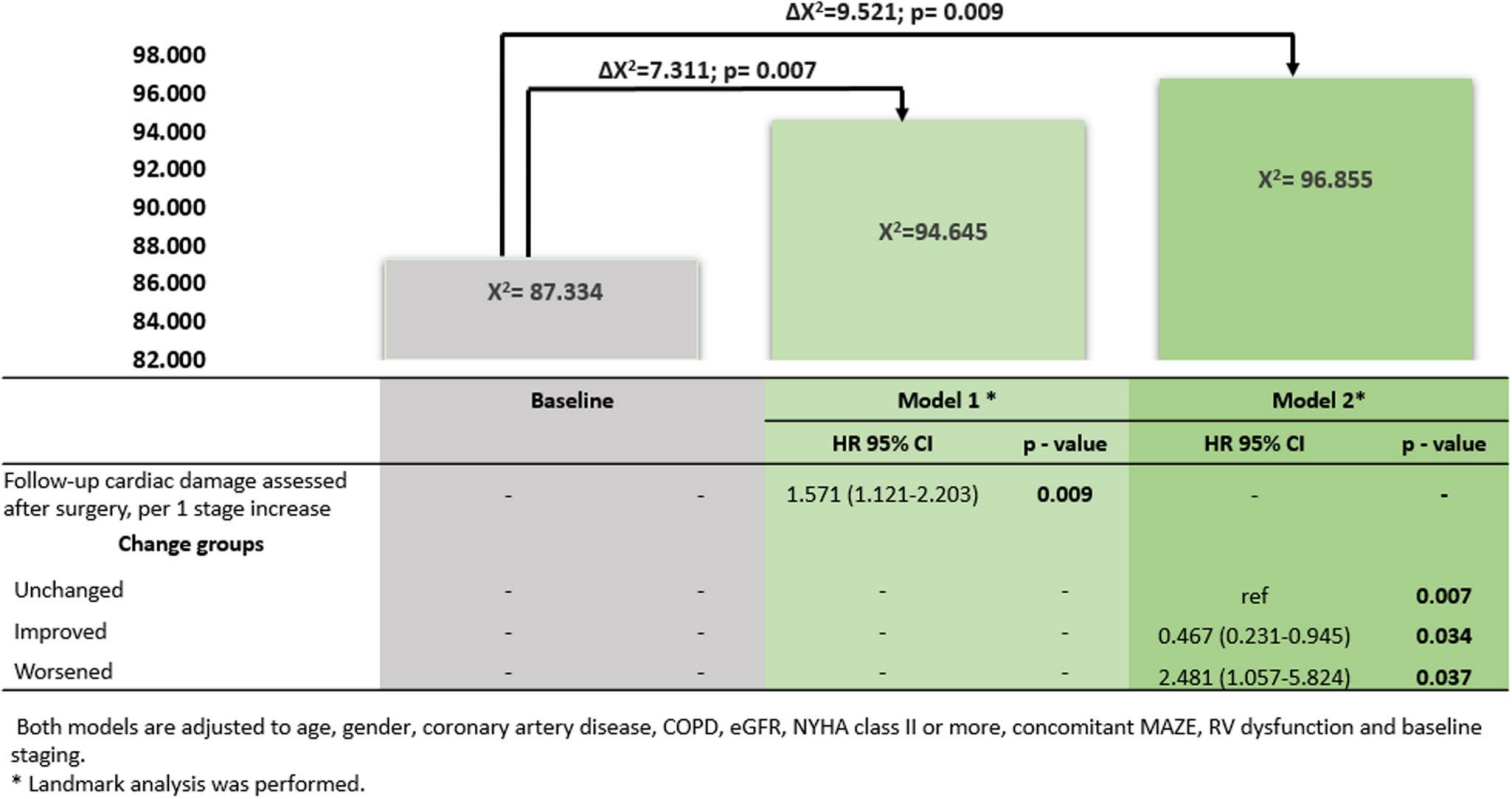
Incremental Prognostic Values of Adjusted Association Between Follow-Up Extra-MV Cardiac Damage and All-Cause Mortality After Surgical Repair. The figure illustrates the incremental prognostic value of assessing the evolution of cardiac damage following MV repair, compared to pre-procedural or follow-up assessments alone. Prognostic models were adjusted for potential confounders, including age, gender, CAD, COPD, eGFR, NYHA class, and pre-procedural RV dysfunction. The results demonstrate that evaluating the change in cardiac damage over time—rather than relying solely on static pre- or post-procedural assessments—provides the highest prognostic accuracy. *Landmark analysis was performed. Abbreviations: CI, confidence interval; COPD, chronic obstructive pulmonary disease; eGFR, estimated glomerular filtration rate; HR, hazard ratio; NYHA, New York Heart Association; RV, right ventricular.Alt text: The graph with 3 bar charts and table underneath represents comparison of 3 prognostic models with follow-up stages and change groups, seperately.

The univariable Cox regression analysis (**Table S5**) showed an association between the following variables and all-cause mortality: age, sex, coronary artery disease, chronic obstructive pulmonary disease, renal function, NYHA class ≥ II HF symptoms, baseline TAPSE, and extra-MV cardiac damage staging at baseline and follow-up. To avoid collinearity, 2 multivariable Cox regression models were performed, one including extra-MV cardiac damage assessed at follow-up and the second one including the evolution of cardiac damage after MV repair (**Figure 3**). After adjusting for potential confounders as well as baseline staging, extra-MV cardiac damage staging assessed at follow-up after MV repair showed an independent association with all-cause death (HR per 1 stage increase 1.571; 95% CI 1.121-2.228, *P* = .007) together with age, sex, coronary artery disease, chronic obstructive pulmonary disease (COPD), estimated glomerular filtration rate (eGFR), NYHA class, concomitant MAZE, RV dysfunction, and baseline staging (**Figure 3**, Model 1). Similarly, also the change in stage groups remained significantly associated with all-cause death after adjusting for other clinical and echocardiographic variables (unchanged as reference group, improved group HR 0.467; 95% CI 0.231-0.945, *P* = .034, and worsened group HR 2.481; 95% CI 1.057-5.824, *P* = .037; **Figure 3**, Model 2). Both models had incremental prognostic value over baseline staging adjusted for potential confounders (Δχ^2^ = 7.311; *P* = .007 in Model 1 and Δχ^2^ = 9.521; *P* = .009 in Model 2) (**Figure 3**). Importantly, **Figure 3** demonstrates that evaluating changes in cardiac damage after MV repair—rather than relying solely on static pre- or post-procedural assessments—yields the highest prognostic accuracy.

## Discussion

In this multicentre study of patients with at least moderate-to-severe PMR undergoing surgical MV repair, extra MV cardiac damage was frequently observed both before and after MV repair. At follow-up after surgery, significant cardiac reverse remodelling was evident, including reductions in LV and LA size, lower pulmonary pressures, and improved valvular function. Based on the cardiac damage staging system, 43% of patients showed improvement, 49% remained stable, and 8% worsened after MV repair. Both the follow-up stage and the change in cardiac damage from baseline were independently associated with all-cause mortality after adjusting for potential confounders. Importantly, assessing the evolution of cardiac damage post-MV repair provided the highest prognostic value, emphasizing the importance of re-evaluating cardiac damage postintervention to improve follow-up risk stratification compared to relying on pre- or post-procedural status alone.

### Impact of MV repair on cardiac remodelling and function

Our study reinforces the concept that the risk profile of patients with PMR can change substantially after MV repair, making static preprocedural assessment potentially misleading. Key components of extra MV cardiac damage—such as pulmonary hypertension, LA dilation, RV dysfunction, and LV enlargement—may regress post-surgery, as it was demonstrated in this multicentre cohort. These changes reflect significant reverse remodelling and can result in a different prognostic profile, highlighting the need for routine post-procedural echocardiographic reassessment of cardiac damage during follow-up. Notably, patients with worsening cardiac damage after repair exhibited worse survival, while those with improvement showed better outcomes, emphasizing the value of evaluating changes rather than relying solely on pre- or post-operative assessments independently.

In alignment with prior studies,^16^^,^^17^^,^^20^^,^^21^ we observed marked regression in extra-MV cardiac damage post-surgery. However, as expected in PMR, LVEF often declined following MV repair. This decline does not indicate true LV systolic impairment but reflects the elimination of low impedance regurgitant flow into the LA due to significant MR. To account for this physiologic shift, we used a postoperative LVEF threshold of <50% to define LV systolic dysfunction—consistent with prior studies and cardiac magnetic resonance data.^9^^,^^16^^,^^17^^,^^21^ Our findings showed a drop in LVEF from 65 ± 8% to 56 ± 8% at 3-12 months, with 23% of patients falling below this threshold (ie, 50%) postoperatively.

LAVI also became significantly smaller post-repair, from 62 to 41 mL/m^2^, indicating reverse atrial remodelling. Since LA dilation is closely linked to AF, we assessed AF prevalence also during follow-up. However, to avoid misclassification, early transient AF episodes probably triggered by surgical stress were excluded from the post-procedural staging classification. This distinction is clinically relevant, as persistent AF—particularly when longstanding—has different prognostic implications and lower likelihood of stable conversion to sinus rhythm postoperatively.^22^^,^^23^

RV function, typically assessed by TAPSE in clinical practice, poses challenges in the post-surgical setting. Tricuspid annular plane systolic excursion often declines after surgery due to pericardiotomy, myocardial stunning, and cardiopulmonary bypass, which may affect longitudinal RV mechanics^8^^,^^18^^,^^24^^,^^25^ without impacting overall RV contractility. Additionally, 30% of patients in our study underwent concomitant TV annuloplasty, further affecting post-procedural TAPSE. Consequently, we excluded TAPSE from our staging classification. However, to account for pre-existing RV dysfunction, all prognostic models were adjusted for baseline TAPSE. This approach ensured accurate risk stratification while avoiding confounding effects from early postoperative alterations in RV mechanics that may not reflect real changes in RV contractility.

### Prognostic implications of cardiac damage evolution after MV repair

Our staging framework builds upon the classification originally proposed by van Wijngaarden et al,^9^ which demonstrated a strong association between preprocedural cardiac damage stage and all-cause mortality in patients with PMR undergoing MV repair. In that study, each stage increment was associated with a 17% increase in the risk of death, independent of clinical variables. While we retained the original staging criteria, we excluded stage 4 (based on TAPSE) due to its unreliability after surgery, largely attributable to the impact of pericardiotomy and other procedural factors on longitudinal RV motion.^25^^,^^26^ Importantly, even after adjusting for baseline staging, preoperative TAPSE, concomitant myocardial revascularization or MAZE procedure (**Figure S3** and **S4**) and also taking into account different MV repair techniques (based on the date when the procedure was performed—**Figure S5**), our study confirmed that follow-up staging remained strongly associated with mortality—emphasizing the added prognostic value of dynamic, post-procedural cardiac damage reassessment.

Crucially, our approach enables comparative, longitudinal risk stratification rather than relying on static pre- or post-operative snapshots. Static assessments may be misleading, as they fail to capture the trajectory of cardiac remodelling—whether towards recovery or deterioration—which carries critical prognostic implications. By stratifying patients based on the evolution of extra-MV cardiac damage (improved, stable, or worsened), we provide a more nuanced and clinically actionable tool for post-MV repair management. Our findings show that patients with improved cardiac profiles post-repair experienced the best outcomes, while those with worsening cardiac damage had significantly poorer survival—highlighting the need to incorporate follow-up echocardiographic and cardiac damage reassessment into routine clinical practice.

Worsening in cardiac damage at follow-up in some patients could be explained by different reasons. First, extra-MV cardiac damage that persists or even worsens after surgery may not be directly related to primary MR and therefore may not be affected by MV repair. Second, some patients may have undergone surgery at a late stage, when myocardial damage had already become irreversible and thus did not improve despite successful repair. A further possibility is that both mechanisms contribute.

While contemporary guideline algorithms and established risk scores such as Mitral Regurgitation International Database Quantitative (MIDA-Q) registry^27^ or newer machine learning models^28^ primarily rely on static baseline characteristics, they often omit key variables such as PASP, TR severity, or RV function. In contrast, our staging approach offers a pragmatic and integrative method that combines both pre- and post-operative information to reflect real-time, comparative, cardiac adaptation and remodelling after MV-repair.

Looking ahead, this dynamic staging concept could be further enhanced by incorporating advanced echocardiographic modalities, such as 3D echocardiography or strain-based parameters, particularly for more accurate assessment of LA, RV, and LV function as well as comparing with different surgical techniques.^29–31^ However, a major strength of our approach is its reliance on conventional echocardiographic measures, which are widely accessible and allow for consistent, reproducible follow-up assessments even in centres without advanced imaging capabilities and experience. This broad applicability enhances the feasibility and clinical translatability of our model. Another notable strength of this study is its multicentre design and large sample size—encompassing over 750 patients—which provides robust statistical power and supports the generalizability of our findings across different healthcare settings. Moreover, the dynamic nature of cardiac damage may have implications for considerations of futility, as cardiac damage is not irreversible and, in most cases, surgery can lead to meaningful improvement even shortly after the operation. Cardiac status is reset after the surgery and this staging system can help defining the intensity of follow-up depending on the severity and evolution of cardiac damage. Future prospective studies should aim to validate this dynamic staging strategy further and explore its integration into guideline-directed post-operative care pathways, possibly alongside advanced imaging when available.

### Limitations

This study has some inherent limitations. First, its retrospective and observational design introduces potential biases, limiting causal inference. Second, selection bias may be present, as patients with incomplete transthoracic echocardiographic data were excluded. Most likely, healthier patients were not followed with echocardiography at our tertiary centres and were instead referred to peripheral hospitals as also suggested in the analysis presented in **Table S1**. In fact, included patients had a higher degree of cardiac damage, as reflected by a greater proportion of concomitant TV surgery and larger LV and LA volumes compared to the excluded ones. Additionally, only all-cause mortality was available as an end-point, and data on other clinical outcomes could not be assessed. However, all-cause death represents a robust end-point, differently from other cardiovascular outcomes that may sometimes be subjective, particularly in retrospective studies.

Finally, while we aimed to evaluate and correct our analysis for pre-operative RV dysfunction assessed with TAPSE, we did not include TAPSE in the staging classification as this parameter has important limitations in detecting RV dysfunction following cardiac surgery. More comprehensive RV and LV assessments (such as with the use of 3D echocardiography or strain-based parameters) may provide more accurate evaluation and should be considered in future studies.

## Conclusion

Extra MV cardiac damage evolves significantly after MV repair, with 43% of patients showing improvement by at least one stage. Both follow-up staging and the evolution of cardiac damage—whether improvement or worsening—were independently associated with all-cause mortality. Notably, assessing changes in cardiac damage postintervention provided the strongest prognostic value, underscoring the importance of systematic post-repair echocardiographic and extra-MV cardiac damage re-evaluation during follow-up to capture the dynamic nature of cardiac remodelling, enabling personalized follow-up after MV repair.

## Author contributions

Rinchyenkhand Myagmardorj contributed to the conception or design of the work; acquisition, analysis, and interpretation of data for the work; drafting of the work; final approval of the version to be published; and agrees to be accountable for all aspects of the work in ensuring that questions related to the accuracy or integrity of any part of the work are appropriately investigated and resolved. Federico Fortuni contributed to the conception or design of the work; analysis and interpretation of data for the work; drafting of the work; revision of the article critically for important intellectual content; and final approval of the version to be published. Valentina Mantegazza, Aniek L. van Wijngaarden, Hoi Wai Wu, Meindert Palmen, Mauro Pepi, Laura Fusini, Manuela Muratori, Benjamin Essayagh, Christophe Tribouilloy, Thierry Le Tourneau, Yohann Bohbot, Hector I. Michelena, and Maurice Enriquez-Sarano contributed to the acquisition of data for the work and final approval of the version to be published. Jeroen J. Bax contributed to the conception or design of the work; revision of the article critically for important intellectual content; and final approval of the version to be published. Nina Ajmone Marsan contributed to the conception or design of the work; analysis and interpretation of data for the work; final approval of the version to be published; and agrees to be accountable for all aspects of the work in ensuring that questions related to the accuracy or integrity of any part of the work are appropriately investigated and resolved.

## Supplementary Material

ezag149_Supplementary_Data

## Data Availability

The data underlying this article will be shared on reasonable request to the corresponding author.
