# Supplementary material for: Extent and Progression of Cardiac Damage in Patients With Primary Mitral Regurgitation Undergoing Surgical Repair
Source: Eur J Cardiothorac Surg. 2026 Apr 10;68(5):ezag149. doi: 10.1093/ejcts/ezag149 (PMC13161561; doi:10.1093/ejcts/ezag149)
Supplement: ezag149_Supplementary_Data [file ezag149_supplementary_data.zip › Supplementary tables.docx]

**Supplementary tables.**

| **Clinical characteristics** | **Excluded**  **(n=982, 56%)** | **Included**  **(n=764, 44%)** | **p-value** |
| --- | --- | --- | --- |
| **Age, years** | 63 (13) | 62 (13) | 0.134 |
| **Male gender, n (%)** | 679 (69) | 536 (70) | 0.648 |
| **Body mass index (kg/m^2^)** | 25.7 (4.1) | 25.0 (3.7) | **<0.001** |
| **Hypertension, n (%)** | 336 (34) | 262 (38) | 0.094 |
| **Diabetes mellitus, n (%)** | 42 (4) | 23 (3) | 0.166 |
| **Coronary artery disease, n (%)** | 262 (27) | 142 (21) | **0.006** |
| **Atrial fibrillation, n (%)** | 194 (20) | 193 (25) | **0.006** |
| **History of smoking, n (%)** | 318 (39) | 206 (34) | **0.035** |
| **Chronic obstructive pulmonary disease, n (%)** | 34 (7) | 27 (5) | **0.044** |
| **NYHA class II or more** | 661 (69) | 524 (73) | 0.056 |
| **Creatinine** | 1.0 (0.3) | 1.0 (0.4) | 0.266 |
| **Concomitant surgery** |  |  |  |
| **CABG** | 153 (16) | 97 (14) | 0.419 |
| **Tricuspid valve surgery** | 230 (23) | 230 (30) | **0.002** |
| **Maze procedures** | 208 (22) | 136 (21) | 0.876 |
| **Echocardiographic characteristics**  **LVEDD, mm** | 56.6 (6.3) | 56.5 (6.9) | 0.837 |
| **LV mass index, g/m^2^** | 125.0 (29.4) | 125.0 (28.2) | 0.977 |
| **LVEDV index, ml/ m^2^** | 72.2 (20.6) | 79.6 (24.1) | **<0.001** |
| **LVESV index ≥ 30ml/m^2^, n (%)** | 125 (28) | 185 (33) | 0.088 |
| **LVEF, %** | 64.7 (7.3) | 65.0 (7.8) | 0.371 |
| **LVEF<60%, n (%)** | 221 (23) | 178 (23) | 0.677 |
| **LA diameter, mm** | 44.8 (7.9) | 45.6 (7.4) | 0.102 |
| **LA diameter ≥ 55mm, n (%)** | 48 (11) | 60 (11) | 0.972 |
| **LA volume index, ml/m^2^** | 59.4 (25.6) | 61.9 (25.1) | **0.043** |
| **PASP, mmHg** | 37.5 (14.7) | 37.2 (13.3) | 0.683 |
| **TR grade > grade 2, n (%)** | 69 (7) | 38 (5) | 0.071 |
| **TAPSE, mm** | 23.4 (4.5) | 23.8 (4.7) | 0.290 |
| **TAPSE ≤17 mm, n (%)** | 43 (10) | 48 (8) | 0.421 |

**Supplementary table 1S.** Comparison of baseline characteristics between included and excluded patients.

Values are mean (SD), or n (%). Statistical significance at the 0.05 level is shown in bold type.

CABG = Coronary artery bypass graft; eGFR = Estimated glomerular filtration rate; LA = Left atrial/atrium; LV = Left ventricular; LVEDD = Left ventricular end-diastolic dimension; LVEDV = Left ventricular end-diastolic volume; LVEF = Left ventricular ejection fraction; LVESD = Left ventricular end-systolic dimension; LVESV = Left ventricular end-systolic volume; NYHA = New York Heart Association; PASP = Pulmonary artery systolic pressure; TAPSE = Tricuspid annular plane systolic excursion; TR = Tricuspid regurgitation.

**Supplementary table 2S.** Baseline clinical characteristics of the study population divided according to the extra-MV cardiac damage assessed after MV surgery (n=764).

|  | **Follow-up staging** | | | | **p-value*** |
| --- | --- | --- | --- | --- | --- |
|  | **Stage 0**  **(n=344, 45%)** | **Stage 1**  **(n=162, 21%)** | **Stage 2**  **(n=217, 29%)** | **Stage 3**  **(n=41, 5%)** |  |
| **Age, years** | 59.5 (12.3) | 59.5 (13.3) | 64.0 (11.3) | 73.5 (10.6) | **<0.001** |
| **Male gender, n (%)** | 225 (65) | 131 (81) | 160 (74) | 20 (49) | **<0.001** |
| **Body mass index (kg/m^2^)** | 24.8 (3.6) | 25.4 (3.8) | 25.2 (3.9) | 24.6 (3.7) | 0.283 |
| **Body surface area (m^2^)** | 1.9 (0.2) | 2.0 (0.2) | 1.9 (0.2) | 1.8 (0.2) | **<0.001** |
| **Hypertension, n (%)** | 105 (35) | 54 (38) | 82 (40) | 21 (55) | 0.082 |
| **Diabetes mellitus, n (%)** | 11 (3) | 6 (4) | 4 (2) | 2 (5) | 0.613 |
| **Coronary artery disease, n (%)** | 58 (19) | 25 (18) | 46 (23) | 13 (35) | **0.105** |
| **Atrial fibrillation, n (%)** | 0 (0) | 0 (0) | 175 (81) | 18 (44) | **<0.001** |
| **History of smoking, n (%)** | 83 (31) | 39 (32) | 75 (39) | 9 (28) | 0.264 |
| **Chronic obstructive pulmonary disease, n (%)** | 12 (5) | 3 (2) | 11 (6) | 1 (4) | 0.519 |
| **NYHA class II or more** | 237 (69) | 89 (65) | 160 (81) | 38 (97) | **<0.001** |
| **Creatinine** | 1.0 (0.2) | 1.1 (0.5) | 1.0 (0.4) | 1.1 (0.4) | **0.003** |
| **eGFR, mL/min/ /1.73 m** | 79.2 (21.9) | 75.3 (19.4) | 76.4 (25.8) | 62.0 (17.5) | **<0.001** |
| **Concomitant surgery** |  |  |  |  |  |
| **CABG** | 44 (15) | 14 (10) | 34 (17) | 5 (13) | 0.353 |
| **Tricuspid valve surgery** | 77 (22) | 48 (30) | 97 (45) | 8 (20) | **<0.001** |
| **Maze procedures** | 23 (8) | 7 (6) | 99 (54) | 7 (19) | **<0.001** |

Values are mean (SD), or n (%). Statistical significance at the 0.05 level is shown in bold type.

CABG = Coronary artery bypass graft; eGFR = Estimated glomerular filtration rate; NYHA = New York Heart Association.

**Supplementary table 3S.** Baseline echocardiographic characteristics (n=764) of the study divided according to the extra-MV cardiac damage assessed after MV surgery.

|  | **Follow-up staging** | | | | **p-value*** |
| --- | --- | --- | --- | --- | --- |
|  | **Stage 0**  **(n=344, 45%)** | **Stage 1**  **(n=162, 21%)** | **Stage 2**  **(n=217, 29%)** | **Stage 3**  **(n=41, 5%)** |  |
| **LVEDD, mm** | 55.6 (6.4) | 60.3 (6.6) | 56.4 (6.8) | 52.1 (7.5) | **<0.001** |
| **LVESD, mm** | 32.9 (5.9) | 37.2 (7.0) | 36.6 (6.9) | 33.7 (6.3) | **<0.001** |
| **LVESD ≥ 40, n (%)** | 50 (15) | 58 (37) | 66 (31) | 7 (17) | **<0.001** |
| **LV mass index, g/m^2^** | 119.2 (25.4) | 131.9 (26.0) | 128.7 (29.2) | 126.8 (39.2) | **<0.001** |
| **LVEDV index, ml/ m^2^** | 78.0 (22.7) | 87.6 (26.6) | 78.7 (23.4) | 64.6 (18.9) | **<0.001** |
| **LVESV index ≥ 30ml/m^2^, n (%)** | 58 (24) | 53 (48) | 67 (38) | 7 (30) | **<0.001** |
| **LVEF, %** | 67.3 (6.3) | 64.0 (7.5) | 62.4 (8.7) | 63.3 (10.3) | **<0.001** |
| **LVEF<60%, n (%)** | 46 (14) | 48 (30) | 72 (34) | 12 (29) | **<0.001** |
| **LA diameter, mm** | 43.8 (6.6) | 45.4 (6.8) | 48.2 (7.8) | 45.1 (9.8) | **<0.001** |
| **LA diameter ≥ 55mm, n (%)** | 12 (5) | 12 (11) | 34 (19) | 2 (9) | **<0.001** |
| **LA volume index, ml/m^2^** | 57.3 (21.1) | 58.4 (20.9) | 71.3 (30.2) | 63.8 (27.6) | **<0.001** |
| **LA volume index ≥ 60ml/m^2^, n (%)** | 126 (37) | 67 (42) | 130 (61) | 17 (43) | **<0.001** |
| **PASP, mmHg** | 34.6 (11.8) | 35.9 (13.7) | 39.6 (13.3) | 49.8 (14.4) | **<0.001** |
| **PASP>50mmHg, n (%)** | 27 (9) | 22 (15) | 35 (17) | 18 (45) | **<0.001** |
| **TR grade > grade 2, n (%)** | 6 (2) | 5 (3) | 19 (9) | 8 (20) | **<0.001** |
| **TAPSE, mm** | 24.9 (4.4) | 23.9 (4.0) | 22.6 (5.2) | 20.6 (4.8) | **<0.001** |
| **TAPSE ≤17 mm, n (%)** | 6 (3) | 4 (3) | 30 (16) | 8 (35) | **<0.001** |

Values are mean (SD) or n (%). Statistical significance at the 0.05 level is shown in bold type.

LA = Left atrial/atrium; LV = Left ventricular; LVEDD = Left ventricular end-diastolic dimension; LVEDV = Left ventricular end-diastolic volume; LVEF = Left ventricular ejection fraction; LVESD = Left ventricular end-systolic dimension; LVESV = Left ventricular end-systolic volume; PASP = Pulmonary artery systolic pressure; TAPSE = Tricuspid annular plane systolic excursion; TR = Tricuspid regurgitation.

**Supplementary Figure 4S.** Evolution of Echocardiographic Parameters after MV repair in patients who did not receive concomitant myocardial revascularization

| **Variables**  **(n=588)** | **Total population who did receive concomitant myocardial revascularization** | | | |
| --- | --- | --- | --- | --- |
|  |  | **Baseline** | **Follow-up** | **p-value** |
| **LVEDD, mm** |  | 56.2 (6.9) | 48.7 (6.6) | **<0.001** |
| **LVESD, mm** |  | 34.5 (6.7) | 33.5 (6.7) | **<0.001** |
| **LV mass index, g/m^2^** |  | 126.2 (28.7) | 103.9 (31.0) | **<0.001** |
| **LVEDV index, ml/m^2^** |  | 75.3 (19.0) | 56.0 (14.3) | **<0.001** |
| **LVEF, %** |  | 64.8 (7.7) | 56.0 (8.1) | **<0.001** |
| **LVEF ≤ 60%, n (%)** |  | 133 (24) | 409 (72) | **<0.001** |
| **LA diameter, mm** |  | 45.3 (7.5) | 39.5 (7.2) | **<0.001** |
| **LA volume index, ml/m^2^** |  | 61.2 (25.4) | 40.0 (16.9) | **<0.001** |
| **PASP, mmHg** |  | 36.6 (12.9) | 25.2 (11.2) | **<0.001** |
| **TAPSE, mm** |  | 23.7 (4.7) | 17.7 (3.0) | 0.346 |
| **TAPSE ≤17 mm, n (%)** |  | 36 (9) | 177 (40) | **<0.001** |
| **MR grade >2, n (%)** |  | 588 (100) | 19 (3) | **<0.001** |
| **Follow-up Staging criteria** | |  |  |  |
| **LVESD ≥ 40, n (%)** | **Stage 1** | 126 (23) | 98(18) | **<0.001** |
| **LVESV index ≥ 30ml/m^2^, n (%)** |  | 126 (31) | 90 (22) | **<0.001** |
| **LVEF ≤ 50%, n (%)** |  | 20 (4) | 120 (21) | **<0.001** |
| **LA diameter ≥ 55mm, n (%)** | **Stage 2** | 44 (11) | 18 (4) | **<0.001** |
| **LA volume index ≥ 60ml/m^2^, n (%)** |  | 218 (43) | 52 (10) | **<0.001** |
| **AF, n (%)** |  | 156 (27) | 155 (27) | 1.000 |
| **PASP>50mmHg, n (%)** | **Stage 3** | 66 (13) | 14 (3) | **<0.001** |
| **TR grade > grade 2, n (%)** |  | 31 (5) | 23 (4) | **0.001** |

Values are mean (SD) or n (%). Statistical significance at the 0.05 level is shown in bold type.

LA = Left atrial/atrium; LV = Left ventricular; LVEDD = Left ventricular end-diastolic dimension; LVEDV = Left ventricular end-diastolic volume; LVEF = Left ventricular ejection fraction; LVESD = Left ventricular end-systolic dimension; LVESV = Left ventricular end-systolic volume; PASP = Pulmonary artery systolic pressure; TAPSE = Tricuspid annular plane systolic excursion; TR = Tricuspid regurgitation.

**Supplementary table 5S.** Univariate Cox regression analysis for evaluating the association of clinical characteristics and staging with all-cause mortality.

| **Univariate analysis** | | |
| --- | --- | --- |
| **Variables** | **HR (95% Cl)** | **p-value** |
| **Age, years** | 1.146 (1.117-1.176) | **<0.001** |
| **Male gender** | 1.023 (0.663-1.579) | 0.918 |
| **Body mass index, kg/m^2^** | 1.060 (1.007-1.116) | **0.026** |
| **Hypertension** | 1.776 (1.172-2.694) | **0.007** |
| **Coronary artery disease** | 2.616 (1.704-4.016) | **<0.001** |
| **Smoking** | 1.286 (0.822-2.011) | 0.271 |
| **Diabetes mellitus** | 0.723 (0.178-2.936) | 0.650 |
| **Chronic obstructive pulmonary disease** | 2.643 (1.319-5.298) | **0.006** |
| **NYHA class II or more** | 2.171 (1.076-4.378) | **0.030** |
| **Creatinine, mg/dl** | 1.896 (1.409-2.550) | **<0.001** |
| **eGFR, mL/min/ /1.73 m** | 0.968 (0.957-0.979) | **<0.001** |
| **TAPSE, mm** | 0.929 (0.883-0.976) | **0.004** |
| **TAPSE (< 17mm)** | 3.075 (1.702-5.556) | **<0.001** |
| **Concomitant surgeries** |  |  |
| **CABG** | 1.896 (1.171-3.069) | **0.009** |
| **Tricuspid valve repair** | 1.116 (0.723-1.722) | 0.621 |
| **MAZE** | 1.918 (1.199-3.066) | **0.007** |
| **Baseline staging, per 1 stage increase** | 1.517 (1.227-1.877) | **<0.001** |
| **Baseline Staging**  **Stage 1 vs Stage 0 (LVEF <60%)** | Ref  1.804 (0.782-4.162) | **0.003**  0.167 |
| **Stage 2 vs Stage 0** | 2.831 (1.472-5.444) | **0.002** |
| **Stage 3 vs Stage 0** | 3.576 (1.742 -7.339) | **<0.001** |
| **Follow-up staging, per 1 stage increase** | 2.075 (1.677-2.568) | **<0.001** |
| **Follow-up staging**  **Stage 1 vs Stage 0 (LVEF <50%)** | ref  1.935 (1.003-3.735) | **<0.001**  **0.049** |
| **Stage 2 vs Stage 0** | 3.253 (1.905-5.552) | **<0.001** |
| **Stage 3 vs Stage 0** | 10.997 (5.797-20.860) | **<0.001** |

CABG = Coronary artery bypass graft; CI = Confidence interval; eGFR = Estimated glomerular filtration rate; HR = Hazard ratio; LVEF = Left ventricular ejection fraction; NYHA = New York heart association; TAPSE = Tricuspid annular plane systolic excursion.
