# Supplementary figures and images for: Extent and Progression of Cardiac Damage in Patients With Primary Mitral Regurgitation Undergoing Surgical Repair
Source: Eur J Cardiothorac Surg. 2026 Apr 10;68(5):ezag149. doi: 10.1093/ejcts/ezag149 (PMC13161561; doi:10.1093/ejcts/ezag149)

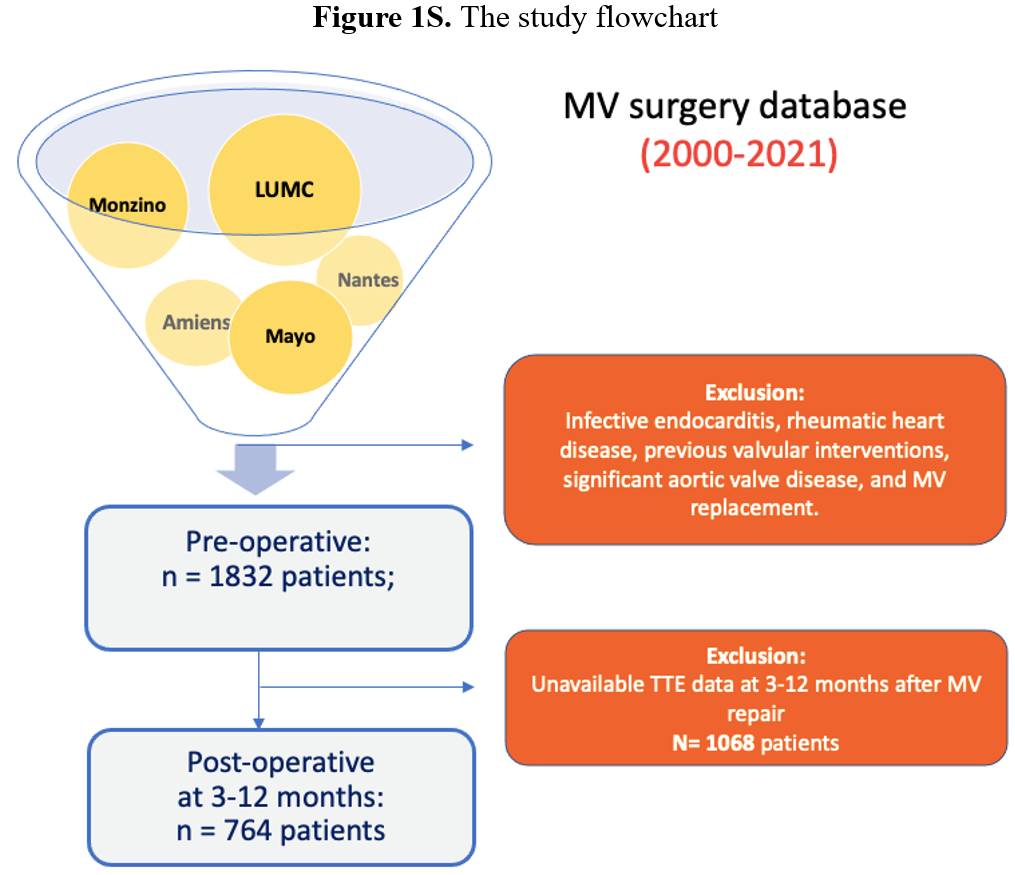


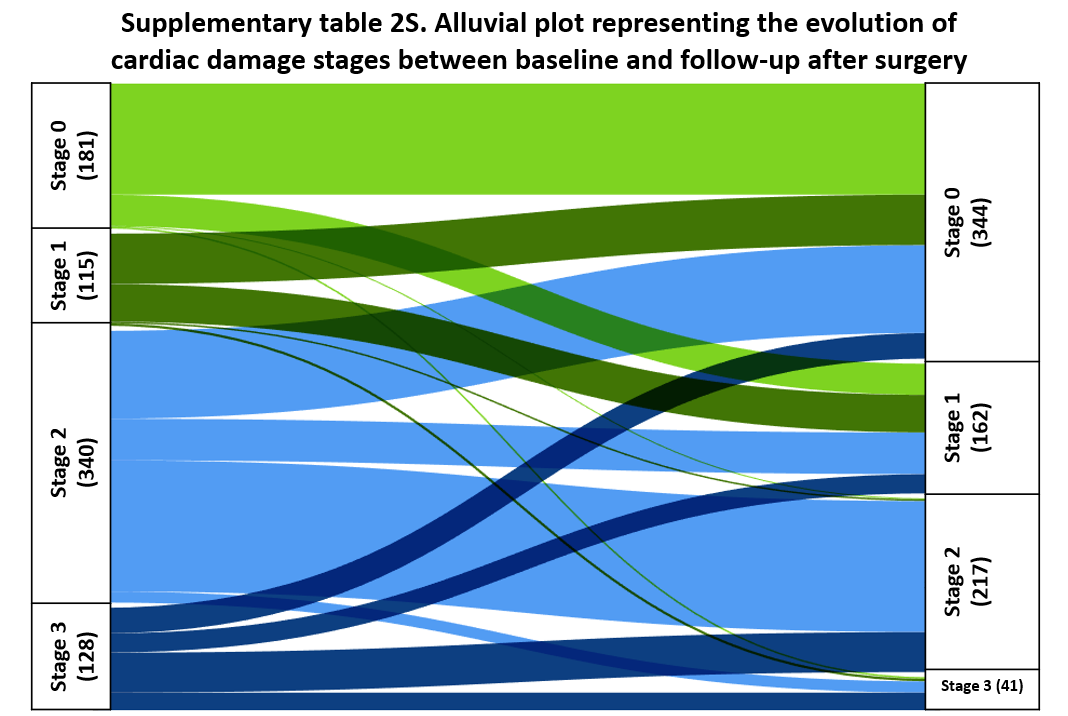


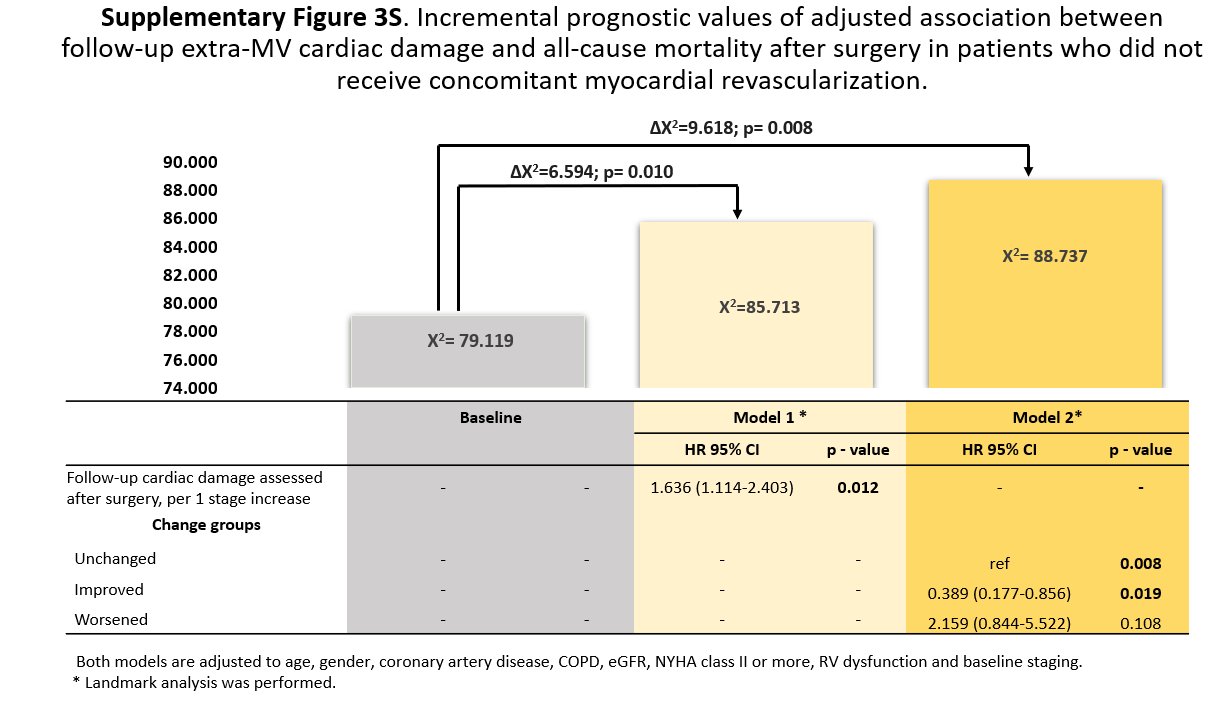

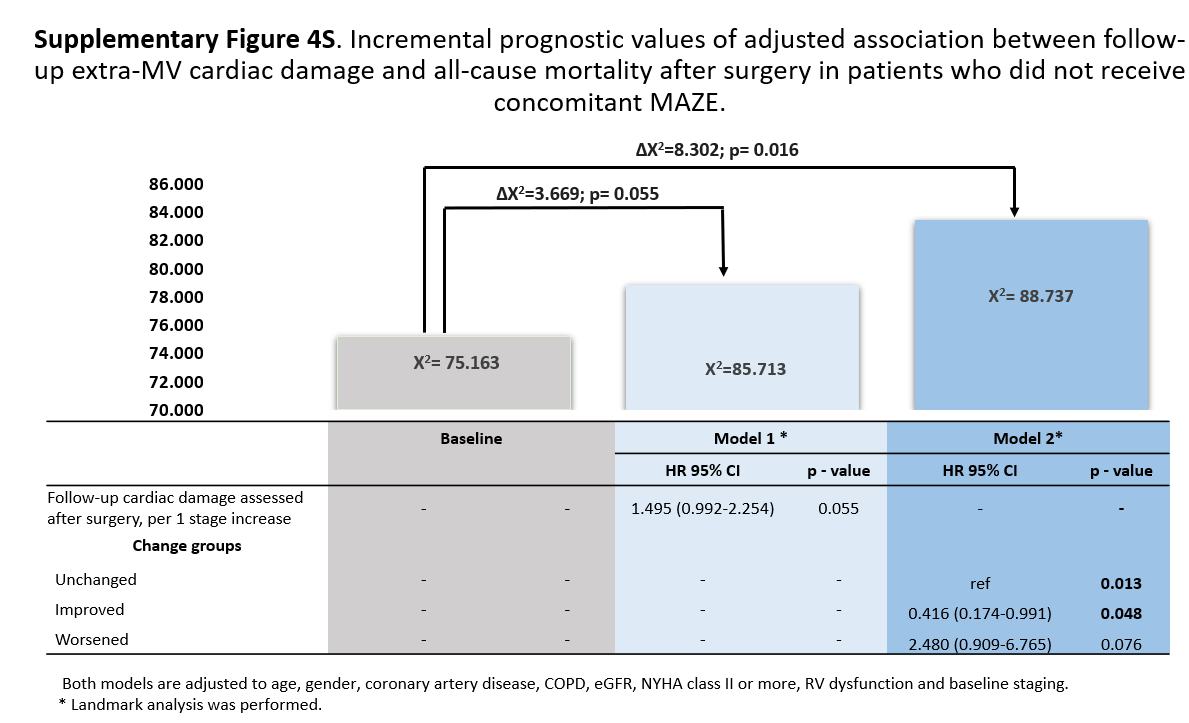

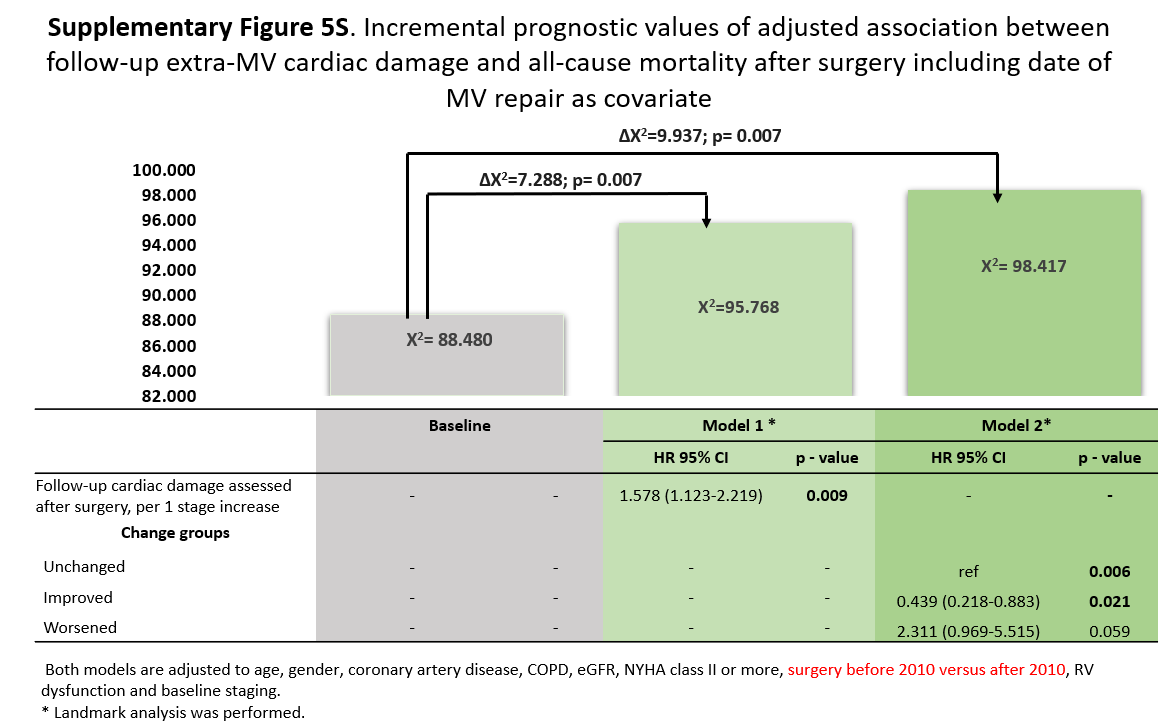

Supplement: ezag149_Supplementary_Data [file ezag149_supplementary_data.zip › Supplementary figures.docx]
